# Transthoracic minimally invasive closure for the treatment of ruptured sinus of Valsalva Aneurysm: a case report

**DOI:** 10.1186/1749-8090-9-27

**Published:** 2014-02-08

**Authors:** Xingxu Cao, Fengwei Zhang, Lingling Wang, Hao Jing, Naiyan Li

**Affiliations:** 1 Department of Cardiovascular Surgery, Linyi People’s Hospital, 27 Jiefang Road, Lanshan District, PO Box: 276000, Linyi, Shandong Province, China

**Keywords:** Aneurysm of the sinus of valsalva, Transthoracic, Closure

## Abstract

Ruptured sinus of the valsalva aneurysm (RSVA ) is a rare cardiac anomaly. Nearly 80% of patients will have symptom when RSVA ruptures into one of the cardiac chambers. The conventional treatment of RSVA is surgical repair under cardio-pulmonary bypass (CPB) or percutaneous catheter closure. Here, we present one case of RSVA undergo transthoracic minimally invasive closure, which is a novel methods for the treatment of RSVA. We describe a thirty four-year-old Asian man with chest pain and palpitation for 3 days. Echocardiography showed that RSVA presented in the right coronary cusp. The sinus opened into the right atrium, and the diameter of the opening was 5 mm. The opening was successfully closed by transthoracic minimally invasive closure with a ventricular septal defect (VSD) occluder.

## Background

RSVA is a rare congenital heart disease, which usually occurs in adolescence or early adulthood. Most patients with RSVA will have symptom when RSVA ruptures into one of the cardiac chambers. Echocardiography can accurately demonstrate the defect and help in establishing the diagnosis. Surgical repair under CPB or percutaneous catheter closure has been the traditional treatment for RSVA. Here, we report the first case of RSVA closed by transthoracic minimally invasive closure with a VSD occluder.

## Case presentation

A thirty four-year-old Asian man was admitted to our department with chest pain and palpitation for 3 days. His past medical history was unremarkable. There was no history of syncope. Clinical examination revealed tachycardia, short breath, high volume pulse with blood pressure of 110/55 mmHg. Auscultation revealed a louder and longer continuous diastolic murmur at the left third intercostal edge. Moist rale were heard in the bilateral lung fields. Echocardiography showed that RSVA was present in the right coronary cusp. The sinus opened into the right atrium, and the diameter of the opening was 5 mm. There was no aortic regurgitation.

We addressed all treatment ways to the patient. To the patience preoperative, including the procedure and complications. This patience worried about cardiopulmonary bypass and the trauma by whole thoracotomy. And also he did not want to undertake the risking of reoperation as the closure failing with percutaneous intervention. After informed consent, patient agreed for the transthoracic closure.

**Figure 1:**
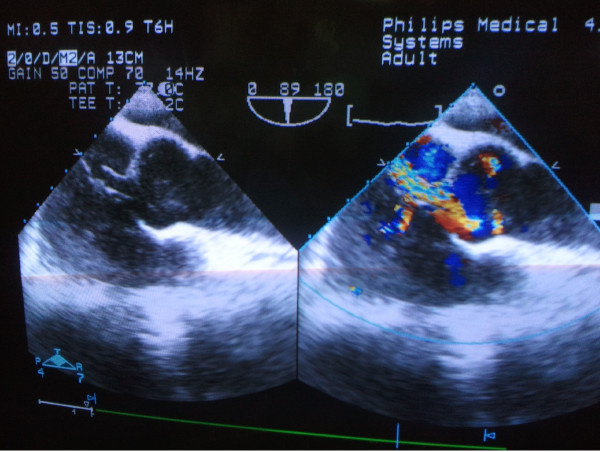
**The maximal diameter of the aortic opening of the RSVA was measured on multi-plane TEE images.** There was no significant aortic valve regurgitation.

**Figure 2:**
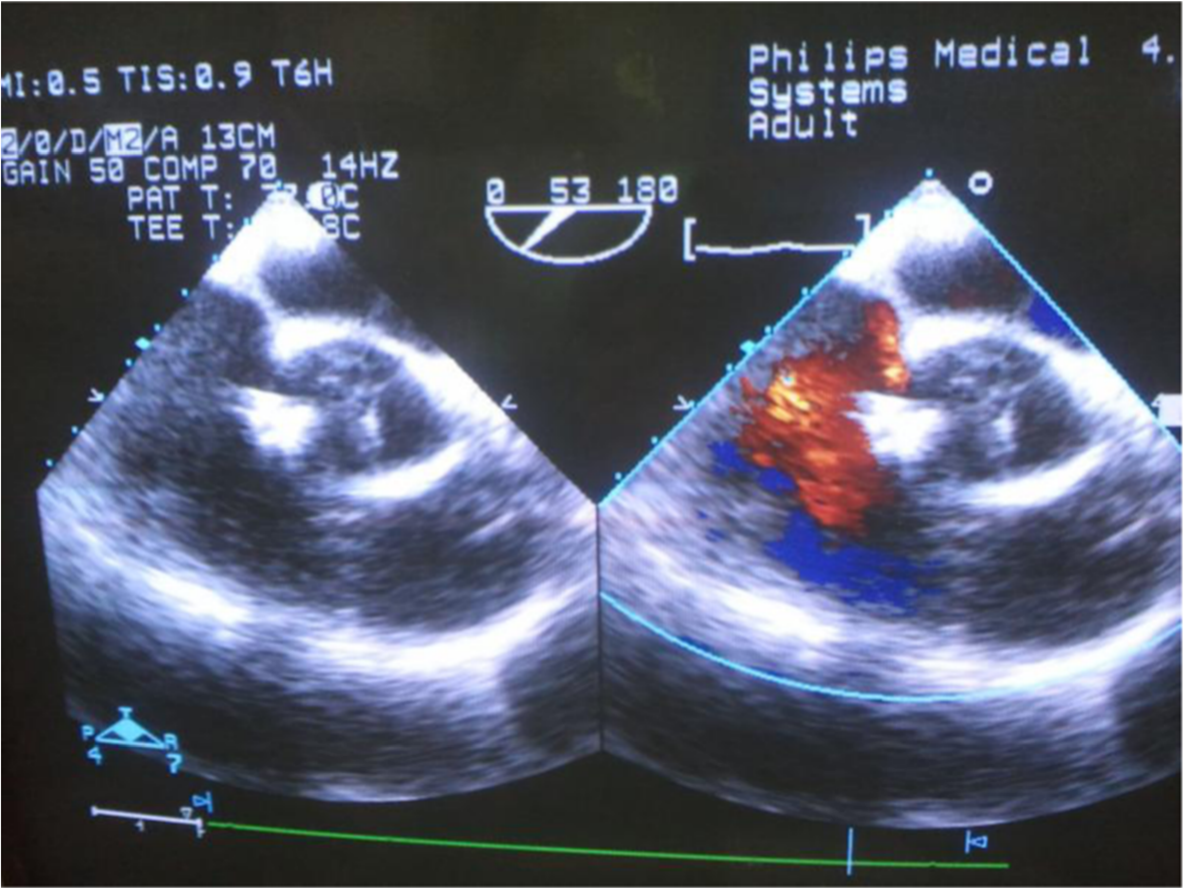
**A VSD occluder was advanced along the sheath.** When the left to right shunt disappeared, the device was deployed. There was not any significant aortic valve regurgitation or residual shunt.

The surgery was performed under general anesthesia with endotracheal intubation. Intraoperative transesophageal echocardiography (TEE) was performed to monitor the procedure. The maximal diameter of the opening of the RSVA was measured by TEE (Figure 1). The distance between the aortic opening site of the RSVA and the right or left coronary ostium was also measured. Sternum upper segment “J form” incision was made to expose the heart. After the pericardium was opened, heparin (1 mg/kg) was administered. Draw a straight line from the opening to the right coronary sinus. The intersection point between the line and ascending aorta was chosen for the puncture point. A guide wire with a soft and floppy end was placed into the right atrium through the rupture opening. Then, an 6-Fr delivery sheath (shape memory alloy material CO, LTD, shanghai, China) was sent into the right atrium along the guide wire. A VSD occluder (shape memory alloy material co., LTD, shanghai, China) 2 mm larger than the maximal diameter of the RSVA opening was implanted along the sheath. The first disc was opened in the right atrium and was pulled back to anchor at the rupture site, then another disc was opened. When the left to right shunt disappeared, the rest of the device was deployed (Figure 2). The whole procedure was guided and monitored by TEE. Aortic valve regurgitation or residual shunt was exclude before detaching the device. After detaching the device from the delivery cable, TEE was repeated to evaluate the position of the occluder, residual shunt and aortic valve function. The patient received aspirin (100 mg per day) for a six months period and follow-up. Transthoracic echocardiography was examined at intervals of 1, 3 and 6 months after discharge. The result was satisfactory. There was no significant complications.

## Discussion

RSVA is a rare congenital cardiac anomaly, which usually occurs in adolescence or early adulthood. RSVA is often caused by congenital weakness of the wall of the sinus. It can lead to progressive dilatation and ultimately rupture into the cardiac chambers or the mediastinum [1]. Patients with the unruptured aneurysm of the sinus of Valsalva usually do not have any symptom, but nearly 80% of the patients will have symptom when RSVA ruptures into one of the cardiac chambers [2]. The conventional treatments of RSVA mainly include surgical repair with patch closure under CPB and percutaneous catheter closure with occluder. Although the mortality is low, the injure of the surgery and cardiopulmonary bypass is significant to patients [3,4]. Percutaneous catheter closure of RSVA was first described by Cullen in 1994, which has been widely used in the treatment of ruptured aortic aneurysm recently [5,6]. But percutaneous catheter closure was complex to transit to conventional sternotomy when closure was failed.

**Figure 3:**
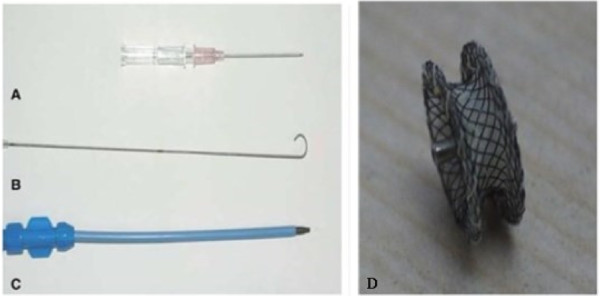
**There were transthoracic minimally invasive closure devices. A** was the puncture needle; **B** was the guide wire with a soft and floppy end; **C** was the livery sheath; **D** was the vsd occluder.

In order to overcome these problems, we applied the novel method of transthoracic minimally invasive closure with VSD occluder to close the opening of the RSVA without CPB (Figure 3). And the early and follow-up outcomes were satisfactory. In our procedure, a “J form” incision was made to reduce the trauma and keep the stability of sternum. Transthoracic closure gets rid of the limitation of vascular conditions and the X-ray. The delivery tract has shorter path and the occluder is easy to release in a more stable position. In addition, we can easily transit to the conventional sternotomy treatment when the closure is failed. Apart from the feasibility of the procedure, caution must be taken to exclude any significant aortic valve regurgitation or residual shunt, which may be associated with hemolysis due to high pressure flow or infective endocarditis.

## Conclusions

In summary, transthoracic minimally invasive closure of RSVA may be another safe and efficient treatment for RSVA patients without significant aortic regurgitation or VSD.

## Consent

Written informed consent was obtained from the patient for publication of this Case report and any accompanying images. A copy of the written consent is available for review by the Editor-in-Chief of this journal.

## Abbreviations

RSVA: Ruptured sinus of the Valsalva aneurysm; VSD: Ventricular septal defect; TEE: Transesophageal echocardiography.

## Competing interests

The authors declare that they have no competing interests.

## Authors’ contributions

XXC and FWZ wrote the draft of the manuscript and obtained the written consent. XXC and LLW performed the literature review and participated in the manuscript writing and helped to the final writing of the paper and gave final approval of the manuscript. HJ and NYL performed the manuscript review and participated in the manuscript revision. All authors have read and approved the final manuscript.
